# Airborne signals of communication in sagebrush: a pharmacological approach

**DOI:** 10.1080/15592324.2015.1095416

**Published:** 2015-09-29

**Authors:** Kaori Shiojiri, Satomi Ishizaki, Rika Ozawa, Richard Karban

**Affiliations:** 1 Faculty of Agriculture; Ryukoku University; Otsu, Japan; 2 Department of Natural Environmental Science; Niigata University; Niigata, Japan; 3 Center for Ecological Research; Kyoto University; Kyoto, Japan; 4 Department of Entomology; University of California; Davis, CA USA

**Keywords:** airborne signals, induced response, plant volatiles, plant communication, sagebrush, 1,8-cineol, β-caryophyllene

## Abstract

When plants receive volatiles from a damaged plant, the receivers become more resistant to herbivory. This phenomenon has been reported in many plant species and called plant-plant communication. Lab experiments have suggested that several compounds may be functioning as airborne signals. The objective of this study is to identify potential airborne signals used in communication between sagebrush (*Artemisia tridentata*) individuals in the field. We collected volatiles of one branch from each of 99 sagebrush individual plants. Eighteen different volatiles were detected by GC-MS analysis. Among these, 4 compounds; 1.8-cineol, β-caryophyllene, α-pinene and borneol, were investigated as signals of communication under natural conditions. The branches which received either 1,8-cineol or β-caryophyllene tended to get less damage than controls. These results suggested that 1,8-cineol and β-caryophyllene should be considered further as possible candidates for generalized airborne signals in sagebrush.

## Introduction

Communication occurs when plants become more resistant to herbivory after they receive volatiles cues emitted by damaged neighbors.^1^ This phenomenon has been reported from a wide diversity of plant species throughout the plant kingdom.^2^ Volatile cues are required although the chemical nature of the volatile cue remains unknown. The volatiles that have been identified have been found to vary depending upon plant species and also genotype for agricultural crops such as maize,^3^ cotton,^4^ wheat^5^ and rice.^6^ Similarly, volatiles emitted by wild plant species such as *Nicotiana*,^7,8^ *Datura*,^9^ *Solanum*^10,11^ have been found to vary among different genotypes. Hybrid poplars released different amounts of monoterpenes and isoprene depending upon their genotype.^12^ It is not known whether the same cues are conserved across the diverse plant species that have been found to respond to volatile cues of damage or whether particular species use unique compounds or combinations as cues.

Several molecular and physiological approaches have been used to attempt to identify airborne cues. For example, when infested lima bean leaves were exposed to terpenes [(*E*)- β-ocimene, (*E*)-4,8-dimethyl-1,3,7-nonatriene (DMNT), (*E*)-4,8,12-trimethyl-1,3,7,11-tridecatetraene (TMTT)] released by other leaves infested with spider mites, their defensive genes were activated.^13^ These results suggested that these terpenes were the volatile cues involved in communication in lima beans. Several green leaf volatiles (GLVs) such as (*E*)-2-hexenal, (*Z*)-3-hexenal and (*Z*)-3-hexenyle acetate have also been implicated as playing a role in communication. These compounds are emitted by many damaged plants and they have been found to trigger plasma membrane depolarization and cytosolic calcium flux in tomato.^14^ When lima bean plants received (*Z*)-3-hexenyle acetate they produced more extrafloral nectar and attracted natural enemies of herbivores such as ants.^15^ In addition, nonanal and methyl salicylate (MeSA) induced resistance in lima beans toward bacterial infections^16^ These results involving lima beans are exceptional in that they were conducted in the field but in general there are few studies that have examined airborne signaling under natural conditions.

Sagebrush (*Artemisia tridentata*) has been well studied and exhibits communication by airborne signals.^17^ Communication that produced induced resistance to herbivore damage occurred between plants that are within 60 cm apart under field conditions^17^ and was stronger between close relatives than between unrelated strangers.^18^ The volatile compounds emitted by individuals of different genotypes are quite variable^19^ although more closely related individuals have more similar volatile profiles (in prep). Volatiles from damaged sagebrush also induce resistance in neighboring individuals of wild tobacco.^17,20,21,22^

Previous work has started to characterize the volatiles produced by damaged sagebrush. Kelsey and coworkers identified 15 volatile monoterpenes from several subspecies of A. tridentata: camphor, thujone, 1,8-cineole, santolina expoxide, *Artemisia* acetate, camphene, *p*-cymene, α-pinene, β-pinene, artemiseole, Artemisia letone, yomogi alcohol, methyl santolinate, arthole, and methacroein.^23^ Kessler and coworkers compared the volatiles emitted by experimentally damaged and undamaged sagebrush foliage and identified 19 compounds.^22^ Of these, (*E*)-β-ocimene and *p*-cymeme emissions were higher in damaged than undamaged plants. In addition, (*E*)-2-hexanal was emitted by damaged sagebrush and was able to prime wild tobacco for greater resistance against herbivores.^22^

In this study we took a pharmacological approach to identifying potential airborne signals used in communication between sagebrush individuals. We asked 2 questions: 1) Which compounds are most commonly emitted by experimentally damaged sagebrush? 2) Which of those compounds causes sagebrush to become more resistant to herbivory when experimentally delivered to receiver plants?

## Results

### Volatile emission

We detected 18 different volatiles emitted by experimentally clipped sagebrush (**Table**). All of these compounds were quite variable among plants in the meadow such that no compound was detected for all 99 plants (e.g., every compound had 0% concentration for at least one plant). Emissions from some individuals were largely composed of β-thujone (as high as 79.7% of the total volatile emission) and others were largely composed of camphor (as high as 61.6%). Two compounds, 1,8-cineole and β-caryophyllene were detected in the emissions of 98/99 plants. Camphene, sabinine, α-thujone, camphor, and germacrene-D were found in a majority of emissions and the other compounds were found in fewer than half of the plants (Table, ratio of detection).

### Field experiment examining the activity of volatile compounds

Based on these results, we selected 4 compounds to test in field experiments: 1,8-cineole, β-caryophyllene, α-pinene and borneol. The first 2 were selected because they were such common constituents of the emissions of almost all individuals (Table). α-pinene was included because it was found in the emissions of 78/99 plants (Table) and because it has been found to increase plasma membrane potential in other plants (Simon et al. 2012). Borneol was included because it was detected in the emissions of 59/99 plants (Table) and because it has been found to have activity as an insect repellent.^24^

Control branches had approximately 30% of their leaves with some herbivore damage by the end of the season in 2011 **(Figure)**. The compounds significantly affected levels of herbivore damage (F _4,144_ = 2.47, P = 0.047). Branches that had been incubated with 1,8-cineole for 24 hours received approximately half this level of damage (LS means difference = 14.7, P = 0.01). Branches exposed to β-caryophyllene tended to receive less herbivory than controls although the difference was marginally not significant (LSMD = 11.1, P = 0.08). Branches exposed to the other volatiles were not statistically distinguishable from the controls (for α-pinene LSMD = 7.0, P = 0.43; for borneol LSMD = 6.6, P = 0.48).

## Discussion

**Figure 1.**
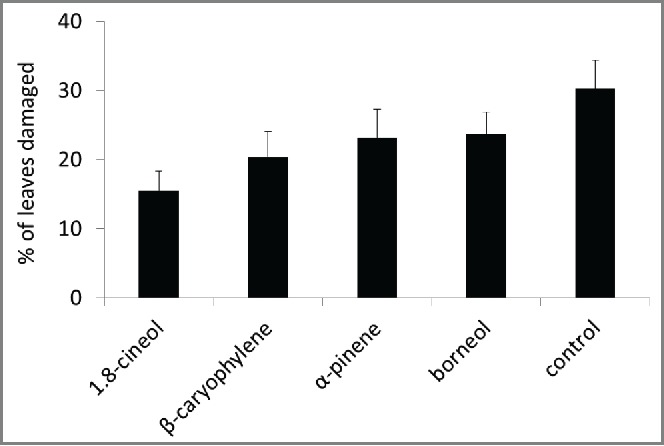
The percentage of leaves with hervibore damage (mean ± SE) accumulated throughout the season plants with filter paper added compounds or with filter paper without added as control.

We previously reported that volatile communication between sagebrush individuals resulted in reduced herbivore damage^17^ Here we found evidence that 2 of the volatiles emitted by damaged sagebrush, 1,8-cineole and β-caryophyllene, may act as signals. These two compounds were emitted by the vast majority of individuals in our study population (**Table 1**). 1,8-cineole was one of 3 compounds that had previously been identified as increasing in response to herbivore damage.^22^ In 2011, branches incubated with 1,8-cineole experienced significantly reduced herbivory and those incubated with β-caryophyllene trended toward a similar effect (**Fig. 1**). We were unable to repeat these results in 2012 although levels of herbivory in that year were uniformly high as over 70% of leaves received damage. These levels of herbivory were considerably higher than we have observed in this system since we started work in 2000 and they might have obscured our ability to detect any treatment effects. Both 1,8-cineole and β-caryophyllene have been reported to have considerable biological activity. In particular, 1,8-cineole has been reported as an antibiotic with inhibitory activity against microorganisms including fungi.^25,26,27,28^ β-caryophyllene is also known to have antibiotic effects.^29,30,31^

**Table 1.** Volatiles from sagebrush

	Range of concentrations (%)	Ratio of detection out of 99 plants
Tricyclene (MS)	0–1.8	35.4
Santolina triene (MS)	0–28.9	5.1
α-Pinene	0–6.3	78.8
Camphene	0–23.6	85.9
Sabinene	0–7.2	86.9
β-Myrcene	0–21.5	13.1
α-Terpinene	0–1.5	35.4
1,8-Cineole	0–27.7	99
γ-Terpinene	0–61.2	53.5
β-Thujone (MS)	0–79.7	79.8
α-Thujone (MS)	0–11.6	97
Camphor	0–61.6	91.9
Borneol	0–4.4	59.6
α-Cubebene	0–0.6	18.2
α-Copaene	0–6.3	40.4
β-Caryophyllene	0–32.4	99
α-Humulene	0–1.7	46.5
Germacrene-D (MS)	0–14.4	86.9

Recently, we have emphasized the specificity in communication among sagebrush individuals; sagebrush responded more effectively to cues from genetically identical clones, from kin, or from individuals of the same chemotype compared to cues from strangers.^2,18,32^ However, even those individuals that received cues from plants that were not genetically or chemically similar responded to become more resistant to herbivory compared to controls. This suggests that plants emit and respond to generalized cues indicating increased risk of herbivory while cues specific to genotype or chemotype may provide more reliable information of risk of herbivory. Our results suggest that 1,8-cineole and β-caryophyllene should be considered further as possible candidates for generalized signals in our system.

More generally, the volatiles emitted by damaged sagebrush have been found to induce or prime resistance to herbivores of tomato and tobacco plants.^33,20,22^ It is possible that 1,8-cineole and β-caryophyllene could be generalized cues that induce resistance in these agriculturally important genera. In the future these candidate compounds will be evaluated as elicitors of resistance in agricultural crops.

## Methods

### Volatile collection and analysis from sagebrush

We collected one branch (approximately 20 cm in length) from each of 99 plants growing naturally in Taylor meadow at the UC Sagehen Natural Reserve, north of Truckee, California (39° 26.7N, 120° 14.7W). All plants were of the same subspecies, *Artemisia tridentata* vaseyana. Branches were kept fresh until volatiles were collected in the lab. On leaf on each branch was cut with scissors and kept in a 300ml Erlenmeyer flask, sealed with parafilm. We collected the headspace volatiles from each leaf for 30 min with SPME fibers (polydimethylsiloxane coating silica fibers, Supelco, Bellefonte, PA). Volatile compounds were analyzed using GC-MS (Agilent Technologies GC model 6890 with an HP-5 MS capillary column 30 cm long, 0.25 mm I.D. and 0.25 um film thickness and a Agilent Technologies MS with a 5973 mass selective detector at 70 eV). The oven temperature of the GC-MS was programmed to rise from 40°C (5 min hold) to 280°C at a rate of 15°C/min. We identified the volatile compounds by comparing their mass spectra to those of a database (Wiley 7N and Wiley 275) and to retention times of authentic compounds. We were not able to compare authentic compounds for several of the volatiles and their identification should be considered as tentative; these are indicated by (MS) in the Table. Some of the compounds were mixtures of different stereochemical isomers and these were not analyzed more fully.

### Field experiment examining the activity of volatile compounds

We conducted a field experiment to examine the potential activity of volatile compounds as inducers of resistance in 2011. Plants were selected along Sagehen Creek (39° 26.7N, 120° 12.9W). We incubated an assay branch with approximately 100 leaves with one volatile compound for 24 hrs. We enclosed this assay branch in a clear plastic bag and placed a square of filter paper (1 cm^2^) to which we added 1 µl of the appropriate compound. This procedure was conducted soon after snowmelt during spring (3 June 2011) when we found sagebrush plants to be most responsive.^34^ Controls were enclosed in a plastic bag with contained a clean filter paper square. Each chemical treatment was replicated on 30 different plants and treated plants were separated by at least 5 m. After 24 hrs, we removed the bag and the filter paper from each branch. We tested 1,8-cineole, α-pinene, β-caryophyllene and borneol plus a control.

We assayed rates of herbivory on the assay branches by counting the number of leaves with any visible damage caused by herbivores at the end of the season (4 October 2011). This measure of herbivory has been used in our previous work in this system and correlates with the percentage of leaf area removed. Our response variable, number of leaves with damage by herbivores, was not normally distributed so we used a logarithmic transformation to meet the assumptions of ANOVA. We analyzed treatment effects caused by exposure to airborne compounds using a GLM (JMP 7.01) on the transformed data although figures present untransformed data. Since we were interested in evaluating treatment effects compared to our control we used Dunnet's test to limit the number of comparisons considered.
